# Solid Lipid Nanoparticles Surface Modification Modulates Cell Internalization and Improves Chemotoxic Treatment in an Oral Carcinoma Cell Line

**DOI:** 10.3390/nano9030464

**Published:** 2019-03-20

**Authors:** Lide Arana, Laura Bayón-Cordero, Laura Isabel Sarasola, Miren Berasategi, Sandra Ruiz, Itziar Alkorta

**Affiliations:** 1 Department of Biochemistry and Molecular Biology, University of the Basque Country (UPV/EHU), Barrio Sarriena S/N, 48940 Leioa, Spain; lbayon95@gmail.com (L.B.-C.); lsarasola008@ikasle.ehu.eus (L.I.S.); berasategi.miren@gmail.com (M.B.); chuenesandra@hotmail.com (S.R.); itzi.alkorta@ehu.es (I.A.); 2 Instituto Biofisika (CSIC, UPV/EHU), Barrio Sarriena S/N, 48940 Leioa, Spain

**Keywords:** solid lipid nanoparticles, phosphatidilethanolamine–polyethileneglycol, controlled drug delivery, cell internalization pathway, cytotoxicity, all-trans retinoic acid

## Abstract

Solid lipid nanoparticles (SLN) present low toxicity, versatility to incorporate both lipophilic and hydrophilic drugs, controlled drug release and they are easy to scale-up. It is well known that the endocytosis pathway by which SLN are taken up and the subsequent subcellular distribution are crucial for the biological effect of the incorporated drug. In addition, interactions between SLN and cells depend on many factors, such as, the composition of nanoparticle surface. In this work different amounts of phosphatidylethanolamine polyethylene glycol (PE–PEG) were added to SLN composed of stearic acid, Epikuron 200 and sodium taurodeoxycholate. Characterization of obtained nanoparticle suspensions were performed by the analysis of particle size, polydispersity index, ζ-potential, cell toxicity and cell internalization pathway. We have observed that the presence of PE–PEG improves active cell internalization of the nanoparticles in an oral adenocarcinoma cell line, reducing non-specific internalization mechanisms. Finally, we have tested the effect of surface coating on the efficiency of incorporated drugs using all-trans retinoic acid as a model drug. We have observed that delivery of this drug into PE–PEG coated SLN increases its chemotoxic effect compared to non-coated SLN. Therefore, it can be concluded that surface modification with PE–PEG improves the efficiency and the specificity of the SLN-loaded drug.

## 1. Introduction

A well-designed drug delivery system (DDS) should be able to improve drug solubility and permeability, avoiding drug degradation or fast elimination. In addition, it is expected to selectively carry a drug into its targeted tissue (or cell type), improving drug efficiency and reducing side effects. Hence, development of novel DDS to achieve the best therapeutic results has become a big challenge for the last decades [1]. In general terms, nanotechnology has abruptly broadened the field of DDS and particularly solid lipid nanoparticles (SLN) have emerged as some of the most promising nanocarriers for controlled drug delivery in the treatment of complex diseases (i.e., cancer or brain pathologies) [2,3].

SLN are submicron-sized dispersions composed of a solid lipid core and a surfactant (and in some cases a co-surfactant), which surrounds the lipid core and helps the assembly of lipophilic components in an aqueous solution. SLN present many advantages in comparison to other drug delivery systems. They are able to incorporate hydrophilic and lipophilic drugs, present no biotoxicity and their production can be easily scaled up [2,4,5]. Because of their solid core, drug release is controlled and they can prevent drug degradation due to solvent contact [6]. Their size and liposolubility enables drug diffusion through some biological barriers, including blood–brain barrier [7,8] and they are not readily taken up by cells of the reticuloendothelial system, so that their filtration in liver or spleen is reduced [9]. Of particular interest is the fact that SLN tend to accumulate preferentially in tumor tissues versus normal tissues due to the so-called “enhanced permeability and retention effect” [10]. Taking together all these attractive characteristics, SLN seem to be a powerful tool for drug delivery to tumors, contributing to improve chemotherapeutic strategies.

Another critical point concerning chemotherapy is the capability of some cancer cells to develop multidrug resistance (MDR). The MDR phenotype observed in many malignancies is frequently associated with the overexpression of P-glycoprotein (P-gp) and other membrane transporters [11]. In this regard, some promising results have shown that SLN are able to avoid this multidrug resistance mechanism, increasing intracellular drug concentration [12] especially when SLN are internalized by endocytosis [13,14,15]. Therefore, SLN internalization pathway is crucial for the efficiency of the drug and the success of the treatment. Additionally, it is known that internalization pathway depends on some factors such as particle size, morphology, composition, hydrophilicity and cell type [16]. Although this type of nanoparticles have demonstrated their capacity to improve drug efficiency, little is known about the mechanism by which SLN enter different cell types. Unfortunately, there is no general rule to predict the internalization pathway for a given SLN composition in a specific cell type. Taking into account the relevance of the uptake mechanisms in the subcellular distribution of a given drug and the implications of the internalization pathway in the efficiency of the treatment, more detailed studies should be addressed in this direction.

Previous investigations have established that a very relevant factor in the clinical use of nanoparticles is their half-life in circulation. It has been reported that surface coating with hydrophilic molecules such as polyethyleneglycol (PEG) can avoid nanoparticle opsonization and uptake by immune cells, reducing nanoparticle clearance by the reticuloendothelial system and improving biological half-life of different DDS [17,18], including SLN [19,20]. Apart from enhancing circulating half-life of different DDS, PEG addition (or PEGylation) can also improve nanocarrier characteristics by stabilization of nanoparticle suspensions, reducing toxicity and improving biocompatibility (reviewed in [21]). Nonetheless, it has to be taken into account that the effect of PEGylation in pharmacokinetic characteristics of the nanoparticles depends on many factors such as PEG molecular weight, density and conformation [21]. For instance, Yang and coworkers demonstrated that PEGylation of nanoparticles should be relatively high-densed to achieve “stealth” properties [22]. Therefore, to obtain a suited DDS for a particular therapeutic treatment, among other factors, the impact of the surface modification on the uptake mechanism of different cells should be studied for each nanoparticle composition. This is particularly relevant when the internalization pathway of the chemotherapeutic drug is fundamental for drug efficiency, as mentioned before.

In the context of controlled drug delivery, apart from endocytic pathways, other drug internalization mechanisms should be taken into account too. In this regard, it has been described that lipophilic cargos can be transferred from nanocarriers to membranes by a direct interaction [23,24]. Hofmann and coworkers described that a temporary interaction between nanoparticles and membranes can facilitate the release of hydrophobic molecules, which eventually localize into lipid droplets [25]. This mechanism, known as “kiss-and-run”, is unspecific and stochastic and should be avoided. It involves the transfer of the cargo from the nanocarrier to any membrane inside the organism. The prevention of a direct interaction between nanoparticles and cell membranes should reduce “kiss-and-run” mechanism. To this aim, modification of nanoparticle surface with PEG could impede a direct interaction between nanoparticle and unspecific cell membrane, reducing unwanted transfer of the cargo. Thus, PEGylation could improve the efficiency of the DDS.

Consequently, to study the effect of PEG coating on SLN characteristics we modified the surface of a promising SLN composition, which had been previously applied to improve the efficiency of a calendula extract treatment [26]. We coated SLN with phosphatidylethanolamine–polyethileneglycol (PE–PEG) at different PEG densities. The best SLN composition based on their physicochemical characteristics was selected and the interactions between SLN suspension and two different human cell lines: an oral squamous carcinoma cell line (SCC-25) and a leukemia derived monocyte cell line (THP-1) were analyzed. We observed that PEG modification did not change interaction between SLN and THP-1 cells, but it was a critical factor in the cell internalization mechanism in SCC-25 cells. Finally, we studied the implication of this surface modification in the efficiency of a drug treatment. For this purpose, we selected all-trans-retinoic acid (ATRA) as a model drug, a well-known chemotherapeutic drug [27], and we studied the efficiency of this drug when incorporated into SLN and into PEG–SLN. As a result, we concluded that PE–PEG coating improves the efficiency of the drug in comparison to not only free ATRA but also when it is vehiculized in SLN.

## 2. Materials and Methods

### 2.1. Materials

Stearic acid (SA) (≥98.5%), sodium taurodeoxycholate hydrate (≥95%), all-trans retinoic acid (≥98%, ATRA), RPMI-1640 medium, DMEM:HAM’S F-12 medium, cytochalasin D, filipin and chlorpromazine were purchased from Sigma-Aldrich (St. Louis, MO, USA). Epikuron 200® (containing about 95% soy phosphatidylcholine, PC) was kindly provided by Cargill (Minneapolis, MN, USA). Amicon Ultra-15 centrifugal filter units were obtained from Millipore (Darmstadt, Germany) and water from a Millipore Milli-Q® ultrapure water purification unit was used. The 1,2-distearoyl-sn-glycero-3-phosphoethanolamine-*N*-[methoxy(polyethylene glycol)-2000] (DSPE-PEG2000) was purchased from Avanti Polar Lipids (Alabaster, AB, USA). Gibco™ Penicillin-Streptomycin-Glutamine (100X), 5-dodecanoylaminofluorescein (C_12_-FITC), octadecyl rhodamine B (R18) and 4,6-diamidino-2-phenylindole (DAPI) were obtained from Thermo Fisher Scientific (Waltham, MA, USA). CytoTox 96® Non-Radioactive Cytotoxicity Assay was obtained from Promega (Madison, WI, USA) and 96-well microtiter plates from Sarstedt (Nümbrecht, Germany).

### 2.2. Preparation of Solid Lipid Nanoparticles

SLN were prepared by the warm microemulsion technique [26]. Briefly, an oil/water (o/w) microemulsion was prepared mixing stearic acid (0.070 mmol), Epikuron 200® (0.014 mmol), sodium taurodeoxycholate (0.066 mmol) and ultrapure water as the continuous phase (11.11 mmol). Stearic acid and Epikuron 200® were first melted together at 80 °C under continuous stirring (1400 rpm). The aqueous phase and the taurodeoxycholate were heated to the same temperature and added to the melted lipid mixture. The mixture was stirred until a transparent, thermodynamically stable microemulsion system was formed. This hot microemulsion was then dispersed into cold water (2–4 °C) using a 1:50 ratio (microemulsion:water, *v*/*v*) under vigorous stirring (14,000 rpm for 10 min) with a SilentCrusher M, Heidolph Instruments (Schwabach, Germany) to form a SLN dispersion. Then, samples were washed three times using Amicon Ultra-15 centrifugal filter units (molecular weight cut-off 100 kDa, Millipore, Billerica, MA, USA) and appropriately diluted in ultrapure water for the following determinations.

Fluorescent probes or PE–PEG were added to the lipid mixture to develop fluorescent SLN (fSLN) or PE–PEG-coated SLN (PEG–SLN). SLN loaded with all-trans retinoic acid were obtained by adding the drug to the melted lipid mix just before aqueous phase addition in order to minimize possible heat-derived drug degradation.

For sterilization purposes, SLN suspensions were passed through 0.22 µm pore filters.

### 2.3. Photon Correlation Spectroscopy

The average diameter and polydispersity index of SLN dispersions were determined by photon correlation spectroscopy (PCS) using a Zetasizer Nano S (Malvern Instruments; Malvern, UK). ζ-potential was determined from electrophoretic mobility using a Zetasizer Nano ZS (Malvern Instruments; Malvern, UK). Each sample was measured in triplicate at 25 °C after dilution with ultrapure water to obtain appropriate concentration.

### 2.4. Transmission Electron Microscopy

Transmission electron microscopy (TEM) analysis was performed using a Philips EM208S microscope in the Analytic and High Resolution Microscopy in Biomedicine Unit of the General Research Services (SGIKER) of the University of the Basque Country (UPV/EHU). TEM samples were diluted 1:20 with ultrapure water and stained with uranyl acetate before analysis.

### 2.5. Incorporation of All-Trans Retinoic Acid in SLN

After forming and washing ATRA-containing SLN dispersions, ATRA–SLN were filtered through 0.45 µm pore filters in order to discard other non-colloidal populations.

Once these SLN were purified, the incorporation of ATRA was determined by measuring the drug content of the obtained suspensions by spectrophotometry. Briefly, suspensions were dried and dissolved in chloroform:methanol (1:1). Sample absorbance was measured at 351 nm and concentration values were obtained using a calibration curve built with ATRA solutions.

Entrapment efficiency (EE) was calculated applying the following formula:

EE = (ATRA amount in SLN)/(Initial ATRA amount) × 100

### 2.6. Cell Culturing

The SCC-25 and THP-1 cell lines used in this work were purchased from American Type Culture Collection (ATCC, Manassas, VA, USA). SCC-25 cells are oral squamous carcinoma cells and they were cultured under standard conditions (humidified atmosphere of 5% CO_2_ at 37 ± 1 °C) in DMEM:HAM’S F-12 supplemented with 400 ng/mL hydrocortisone, 10% heat-inactivated fetal bovine serum (FBS), 50 mg/L penicillin-streptomycin and 0.1 g/L L-glutamine.

THP-1 cells, a human monocytic cell line, were cultured under standard conditions in RPMI-1640 medium supplemented with 10% FBS, 50 mg/L penicillin-streptomycin and 0.1 g/L L-glutamine.

For in vitro experiments with cells, SLN were added in serum-free medium in order to avoid protein adsorption related phenomena [28,29,30] and to obtain a more controlled system to observe interactions between SLN and cells.

### 2.7. In Vitro Cell Cytotoxicity Assay

Cytotoxicity of SLN preparations was tested in human THP-1 and SCC-25 cells by measuring the release of the cytosolic enzyme lactate dehydrogenase (LDH). The quantitative measurement of LDH activity was performed using the CytoTox 96® Non-Radioactive Cytotoxicity Assay according to manufacturer’s instructions (Promega, Madison, WI, USA). In order to obtain total cell cytotoxicity (0% viability) cells were lysed using 0.8% Triton X-100. Untreated cells were used as negative controls. Cytotoxicity (%) of applied treatments was calculated using the following formula:

(1) $$Cytotoxicity\;\left(\%\right)=\frac{\left(sample\;absorbance-control\;absorbance\right)}{\left(lysed\;cell\;absorbance-control\;absorbance\right)}\times 100$$

To establish CC_50_ dose (the SLN concentration that kills 50% of cell population), four-parameter logistic nonlinear regression analyses were performed. Cytotoxicity dose-response curves were plotted using GraphPad Prism 5 software (GraphPad Software Inc., San Diego, CA, USA). The curve equation was calculated applying the following formula:

(2) $$Y=\frac{100}{\left(1+10^{(\left(logCC50-x\right)*HillSlope}\right)}$$

### 2.8. Cell Incorporation Assay by Flow Cytometry

5-dodecanoylaminofluorescein (C_12_-FITC) probe was used in flow cytometry experiments because it is a useful tool to distinguish between uptaken and internalized fluorescent signal in cell incorporation experiments. Trypan-blue can quench C_12_-FITC emitted fluorescence but it cannot enter live cells [31]. Therefore, in a cell suspension with trypan-blue only fluorescence signal of cell internalized fluorescein can be detected. Fluorescence intensity of samples treated with trypan blue was considered as fluorescence from internalized fSLN and fluorescence of samples without trypan blue treatment was considered as cell uptake (attached and internalized fSLN).

SCC-25 cells were seeded in 6-well plates at 1.5 × 10^5^ cells/well cell density. The next day, cells were washed and incubated in serum-free medium. Appropriate concentrations of fSLN suspensions were added and cells were further incubated during different time periods. Then, cells were washed and harvested by trypsinization. THP-1 cells were seeded in 24-well plates at 10^5^ cells/well in serum-free medium. Appropriate concentrations of fSLN suspensions were added and cells were further incubated during different time periods. Cells were then harvested and washed by centrifugation (5 min, 500× *g*).

To achieve endocytosis inhibition assays, cells were incubated with the corresponding inhibitory drugs during half an hour before the addition of fSLN suspension.

Geometrical mean of the fluorescence intensity of cell populations was measured by flow cytometry with an air-cooled 488 nm argon–ion laser (FACSCalibur, BD Biosciences; San Jose, CA, USA) and CellQuest software (Becton Dickinson; Franklin Lakes, NJ, USA), according to the manufacturer’s instructions.

### 2.9. Cell Incorporation Assay by Confocal Microscopy

For confocal microscopy experiments fSLN were obtained by adding octadecyl rhodamine B (R18) to the lipid mixture. SCC-25 cells were seeded on 12-mm glass coverslips that were placed inside 24-well plates at 10^4^ cells/well cell density. The next day, cells were washed and incubated in serum-free medium. fSLN suspensions were added and cells were further incubated during different periods of time. At the given time, cells were washed and fixed for 30 min using paraformaldehyde (4% in phosphate buffered saline (PBS)). After washing the cells, nuclei were stained using 0.5 µg/mL DAPI and coverslips were mounted using ProLong™ Glass Antifade Mountant (Invitrogen, Walthan, MA, USA). Images were obtained using an inverted confocal fluorescence microscope (Nikon D-ECLIPSE C1, Nikon Inc., Melville, NY, USA) with a total internal reflection fluorescence ×60 oil immersion objective. The excitation wavelengths used were 488, 561, and 635 nm, and emitted fluorescence was recorded using band pass filters of BP515, BP593, and a long pass filter of LP650, respectively. Fluorescence images were processed with ImageJ software (National Institutes of Health, Bethesda, MD, USA).

### 2.10. Statistical Analysis

Statistical analysis was performed using the two-tailed, paired Student’s t-test, where *p* < 0.05 was considered to be significant (GraphPad Prism software, San Diego, CA, USA).

## 3. Results

### 3.1. Development and Characterization of PE–PEG Coated SLN

In order to analyze physicochemical characteristics of PEG–SLN we developed different SLN suspensions obtained by adding different amounts of PE–PEG. For this purpose, we substituted a percentage of Epikuron 200 (phosphatidylcholine, PC) with PE–PEG molecules in the initial lipid mixture of the microemulsion formation. Therefore, 1% PE–PEG means that 1% of PC moles have been substituted with the same moles of PE–PEG.

**Figure 1:**
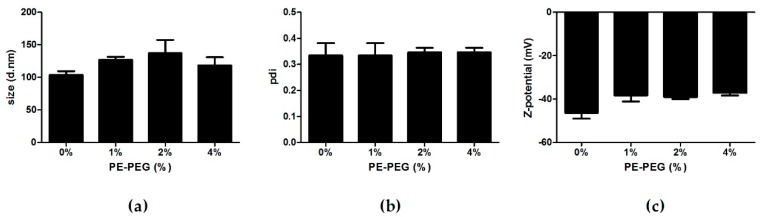
Particle size, polydispersity index and ζ-potential values of solid lipid nanoparticles (SLN) coated with different percentage of phosphatidylethanolamine polyethylene glycol (PE–PEG). (**a**) Particle size, (**b**) polydispersity index (pdi) and (**c**) ζ-potential values of different SLN were obtained by Photon Correlation Spectroscopy. Results are the mean ± SEM of four independent experiments.

We prepared four different nanoparticle suspensions (0, 1, 2 and 4% of PE–PEG) and determined their size, polydispersity (pdi) and ζ-potential by photon correlation spectroscopy. Covering SLN with PE–PEG marginally increased nanoparticle size and slightly decreased ζ-potential of nanoparticle suspensions with 2% and 4% of PE–PEG (Figure 1).

It has been reported that PEG coating increases stability of developed nanoparticle suspensions [32,33]. In order to test this feature we stored different suspensions of nanoparticles in distilled water at 4 °C and we tested the main nanoparticle characteristics at different time points during 1 week. We observed no significant differences in size, polydispersity (pdi) and ζ-potential of the SLN, concluding that PE–PEG coating did not affect nanoparticle stability in these storage conditions (data not shown).

Next, we analyzed nanoparticle morphology by transmission electron microscopy and we observed a similar morphology and size in coated and non-coated SLN suspensions (Supplementary Figure S1).

**Figure 2:**
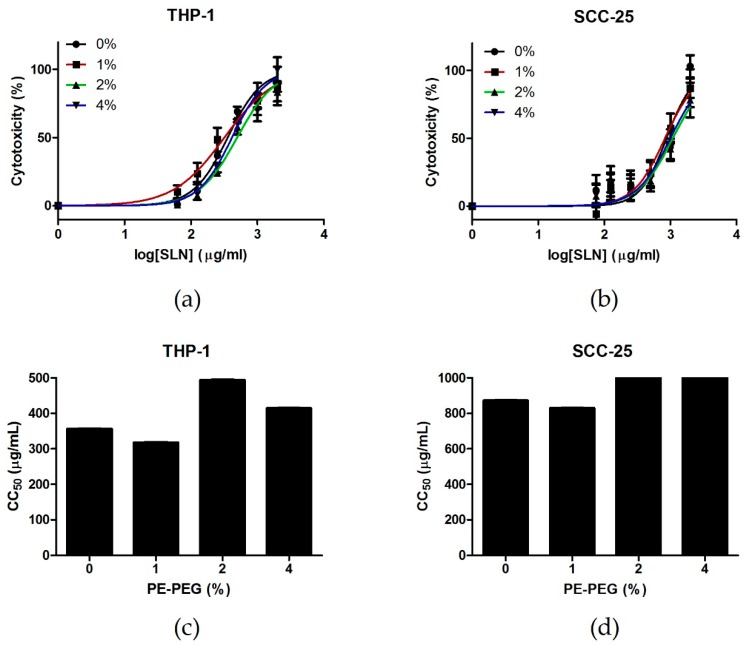
Cytotoxicity of different SLN suspensions in THP-1 and SCC-25 cell lines. (**a**) THP-1 cells were seeded into 96-well culture plates at 2 × 10^4^ cells/well. Then, different concentrations of non-coated SLN (0%) (●) or SLN coated with different percentages of PE–PEG (1% (■), 2% (▲) or 4% (▼)) were added to cell culture. They were incubated for 24 h and cell toxicity was determined by CytoTox 96® Non-Radioactive Cytotoxicity Assay. Cell toxicity (%) was defined as mentioned in Materials and Methods. Results are the mean ± SEM of three independent experiments performed in triplicate. Dose-response curves were plotted using GraphPad. (**b**) SCC-25 cells were seeded into 96-well culture plates at 10^4^ cells/well. The next day, different concentrations of non-coated SLN (0%) (●) or SLN coated with different percentages of PE–PEG (1% (■), 2% (▲) or 4% (▼)) were added to cell culture and cells were further incubated for 24 h. Cell cytotoxicity was determined by CytoTox 96® Non-Radioactive Cytotoxicity Assay. Cell viability (%) was defined as mentioned in Materials and Methods. Results are the mean ± SEM of five independent experiments performed in triplicate. Dose-response curves were plotted using GraphPad. (**c**) CC_50_ of different SLN suspensions were obtained from dose-response curves in THP-1 cell culture. (**d**) CC_50_ of different SLN suspensions were obtained from dose-response curves in SCC-25 cell culture.

It is well known that PEG coating reduces cytotoxicity of different DDS [34,35]. In order to study the effect of PE–PEG coating, we tested cell cytotoxicity of different SLN suspensions performing CytoTox 96® Non-Radioactive Cytotoxicity Assay in two different cell lines: a human monocytic cell line THP-1 and a human epithelial cell line SCC-25. We observed that PEGylation reduced the cytotoxicity of SLN cytotoxicity in both cell lines. Moreover, although the pattern was different, the CC_50_ increased from 0% to 2% PE–PEG in both cell lines. Further increment was not observed for 4% PE–PEG coating (Figure 2).

### 3.2. Incorporation of PEG–SLN in Cell Culture

Extensive analysis of cell incorporation pathway is essential to understand controlled drug delivery, as this process determines drug fate inside the cell. To do so, fluorescent probe incorporation can be analyzed by flow cytometry or confocal microscopy, measuring fluorescence intensity of each cell in the culture. For this purpose, we incubated SCC-25 and THP-1 cells with fSLN and cell uptake was defined as the total fluorescence intensity (coming from cell surface and inside the cells) and cell internalization was the portion of the fluorescence signal coming from inside the cells. 

In order to determine optimal incubation time and nanoparticle concentrations for internalization pathway analysis, we performed concentration- and time-courses with non-coated fSLN by flow cytometry. 

**Figure 3:**
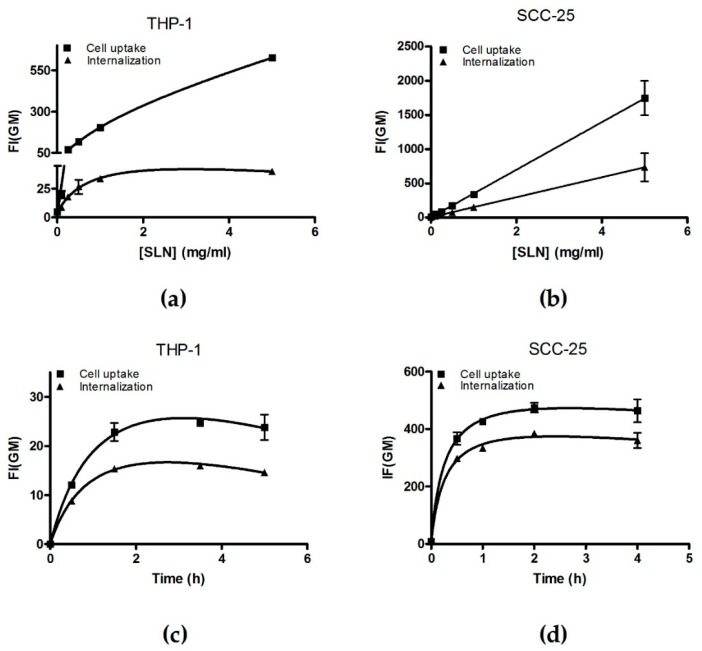
Non-coated fSLN incorporation is concentration and time dependent. THP-1 cells were seeded at 10^5^ cells/mL and (**a**) treated with different concentrations of fSLN for 2 h or (**b**) treated with 0.25 mg/mL of fSLN for different incubation periods. SCC-25 cells were seeded at 1.5 × 10^5^ cells/well in 6-well plates and incubated overnight. Next day, cells were washed and treated with different concentration of fSLN for 2 h (**c**) or incubated with 0.25 mg/mL fSLN for indicated periods of time (**d**). Then, cells were washed in PBS and collected in order to measure GeoMean of the fluorescence intensity (FI(GM)) of incorporated C_12_-FITC by flow cytometry. Samples measured without trypan blue are indicated as cell uptake (■) and samples measured with trypan-blue are indicated as probe internalization (▲). Results are the mean ± Range of two independent experiments. Total binding saturation curve was calculated in order to facilitate data reading.

Analysis of non-coated fSLN uptake by flow cytometry indicated that fSLN incorporation is concentration and time dependent for THP-1 cells. We observed that fSLN cell internalization was saturated at 0.5 mg/mL concentration and after 1.5 h of incubation (Figure 3a,b). When the same analysis was performed for SCC-25 cells again cell internalization was saturated after 1.5 h of incubation but concentration effect remained lineal (Figure 3c,d). In order to characterize interactions between cells and fSLN, measurements must be performed at a dynamic range, so that the differences are more obvious. Therefore, we decided to work at the range of fSLN concentration and incubation time were cell internalization remains linear. These optimal conditions were 0.2 mg/mL of fSLN and 1 h of incubation, for both cell lines.

#### 3.2.1. PE–PEG Coating Improves Active fSLN Internalization Mechanisms in SCC-25 Cells but not in THP-1 Cells

**Figure 4:**
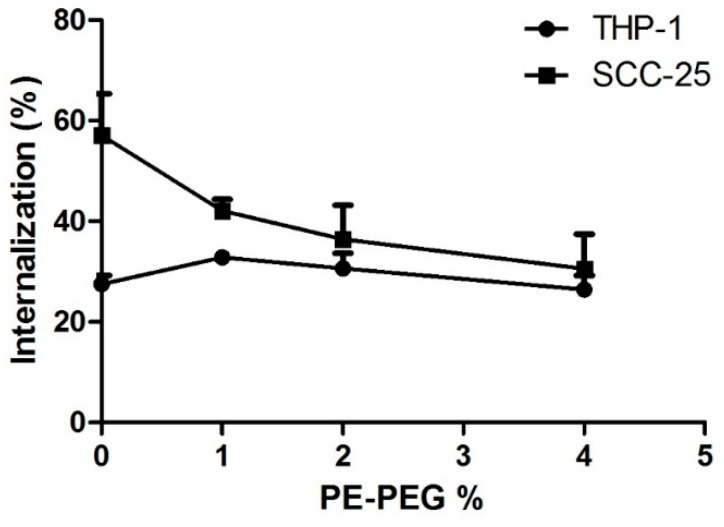
PEG–SLN internalization in different cell lines with ATP depletion treatment. Cells were pre-incubated for 1 h in the presence of 20 mM of sodium azide and 10 mM of 2-deoxyglucose. Then, indicated SLN suspensions were added and cells were further incubated for 1 h. Then, cells were washed and trypan blue was added in order to measure internalized SLN fluorescence. Data are shown as percentage of fSLN internalization relative to the values of internalization in the absence of sodium azide. Results are the mean ± SEM of five independent experiments.

As mentioned before, SLN incorporation pathway must be determined to evaluate SLN suitability as a DDS for chemotherapeutic drugs. Because endocytosis is an energy dependent internalization mechanism, we wanted to study cell internalization of different nanoparticle suspensions in the presence or absence of ATP. For this purpose, we analyzed cell internalization of fSLN in an ATP depleted condition, using sodium azide treatment. We observed that ATP depletion affected to both cell lines but in a different extent (Figure 4). In THP-1 cell line, fSLN internalization was almost blocked in the presence of azide for all tested SLN suspensions. Intriguingly, the inhibition of cell internalization by sodium azide was PE–PEG dependent for SCC-25 cells. Increasing PE–PEG percentage enhanced the effect of ATP depletion, indicating that PE–PEG coating improves active (ATP dependent) cell internalization pathways. Few differences in the ATP depletion effect were observed between 2% and 4% of PE–PEG; therefore, at this point we selected 2% PE–PEG SLN suspension in order to compare SLN with or without PE–PEG coating in SCC-25 cells.

#### 3.2.2. PE–PEG-Coating Does Not Alter Intracellular Distribution of SLN in SCC-25 Cells

**Figure 5:**
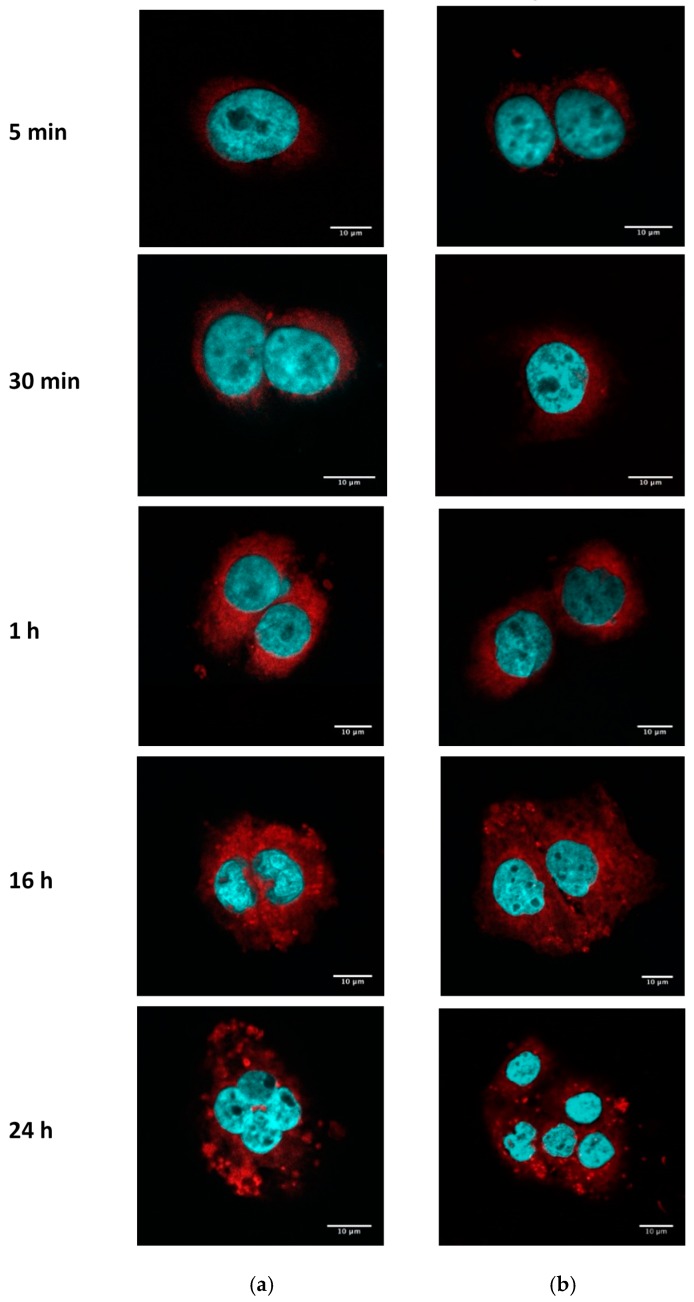
Internalization of fSLN and fPEG–SLN in SCC-25. SCC-25 cells were seeded in cover glasses at 2 × 10^4^ cells/well. The next day, cells were washed and incubated with 0.2 mg/mL of fPEG–SLN (**a**) or fSLN (**b**) suspensions. Fluorescence intensity of R18 (red) was analyzed by confocal microscopy and nuclei were stained with DAPI (blue). Scale bar: 10 µm.

Taking into account the impact of PE–PEG coating on cell internalization in SCC-25, we wondered whether the subcellular distribution pattern could vary depending on the surface of the nanoparticles. We analyzed by confocal microscopy the fluorescence intensity of fSLN with or without surface modification. We observed no differences between the subcellular distribution of both nanoparticle suspensions by this method (Figure 5). In both cases, nanoparticles were internalized at similar time range and cell distribution of fSLN can be observed in the cytosol.

#### 3.2.3. PE–PEG-Coating Affects Cell Internalization Pathway of fSLN in SCC-25 Cells

We next wondered whether PE–PEG modification affected SLN incorporation pathway. Endocytosis can be divided into two main categories: phagocytosis (accomplished by professional phagocytes) and pinocytosis. Pinocytosis can be dived into four different mechanisms: clathrin-mediated endocytosis, caveolae-mediated endocytosis, clathrin- and caveolae-independent endocytosis or macropinocytosis. In order to understand the internalization mechanism involved in fSLN uptake, we incubated cells with selective pharmacological inhibitors of the aforementioned internalization pathways. Filipin (5 µg/mL) inhibits caveolae-mediated endocytosis [15], cytochalasin D (5 µg/mL) inhibits macropinocytosis and phagocytosis [36] and chlorpromazine (5 µg/mL) inhibits clathrin-mediated endocytosis [37].

**Figure 6:**
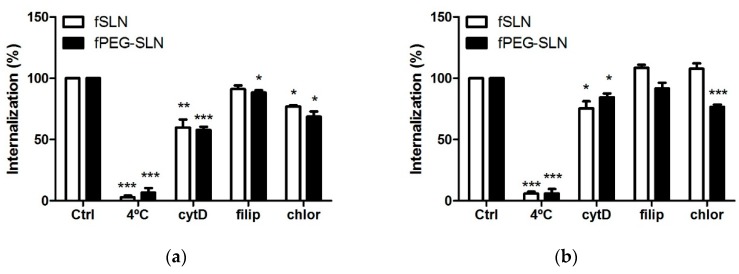
Effect of PE–PEG-coating in fSLN internalization pathway. (**a**) THP-1 cells were seeded at 10^5^ cells/mL and pre-incubated with vehicle (Ctrl), cold treatment (4 °C), 5 µg/mL cytochalasin D (cyt D), 5 µg/mL filipin (filip) or 5 µg/mL chlorpromazine (chlor) for 30 min. Then, cells were treated with 0.2 mg/mL of indicated SLN suspensions and further incubated for 1 h. After that, cells were washed with PBS and fluorescence intensity of internalized C_12_-FITC was measured by flow cytometry; (**b**) SCC-25 cells were seeded at 1.5 × 10^5^ cells/well in 6-well plates. The next day, cells were exposed to the same treatment and they were harvested with trypsin in order to measure their fluorescence intensity by flow cytometry. Data are shown as percentage of intensity of fluorescence related to the values of internalization of C_12_-FITC at 37 °C (100%). Results are the mean ± SEM of four independent experiments. (* *p* < 0.05; ** *p* < 0.01; *** *p* < 0.001).

THP-1 is a human monocytic cell line, which means that these cells can internalize SLN using phagocytosis. This cell line has been used to determine the ability of PEG-coating to abolish phagocytosis of nanoparticles [22]. In our hands, internalization in THP-1 cells of SLN suspensions with or without PEG coating showed same inhibition pattern. Their internalization was inhibited mainly by cytochalasin D and some inhibition was also observed in chlorpromazine treated cells (Figure 6a). Therefore, in THP-1 cells phagocytosis seemed the main internalization pathway and there was some SLN internalization in a clathrin-dependent endocytosis pathway. 

For SCC-25 cells, the internalization mechanism for PEG-coated or non-coated SLN was different, as the effect of endocytosis inhibitors was also different. Internalization of non-coated SLN was hardly inhibited by applied endocytosis inhibitors. Cytochalasin D was the only inhibitor able to reduce cell internalization of non-coated SLN. Thus, macropinocytosis seemed to be the main endocytosis mechanism for non-coated SLN. For PEG–SLN, the main internalization inhibition was obtained with chlorpromazine treatment and to a minor extent with cytochalasin D. Therefore, clathrin-mediated endocytosis was the major mechanism for the internalization of PEG–SLN (Figure 6b). From our results we concluded that for SCC-25 cells PEG-coating changed internalization pathway, from a passive to an active endocytosis mechanism. Thus, applying PEG-coating did not inhibit phagocytosis of SLN in THP-1 cells but it was relevant for the internalization mechanism of SLN in epithelial cells.

### 3.3. PEG Coating Improves the Efficiency of ATRA-Loaded SLN Treatment

In order to study the effect of surface coating in the efficiency of a delivered drug we analyzed the cytotoxic effect of a well-known drug able to suppress tumor cell growth, all-trans retinoic acid (ATRA) [38]. This compound is a Vitamin A derivative that is able to regulate cell proliferation and stimulate cell differentiation in immune cells, and it has been reported to inhibit cell proliferation in SCC-25 cells [39]. Unfortunately, this is a very hydrophobic substance so that organic solvents or DDS are needed in order to add it to cell cultures [40]. Therefore, incorporation of ATRA in SLN could be a good strategy to avoid the application of organic solvents while improving drug efficiency.

#### 3.3.1. ATRA Can Be Easily Loaded into SLN Using an Organic Solvent-Free Method

First, we prepared SLN suspensions adding different amounts of initial ATRA (0.5, 1, 2, 3 mg). We observed that ATRA addition significantly increased SLN mean diameter size. Nevertheless, all developed SLN suspensions presented physical characteristics in the range for drug delivery purposes (Supplementary Figure S2).

We also determined drug entrapment efficiency (EE) and the total amount of incorporated ATRA. We observed that initial 1 mg ATRA addition presented the best entrapment results since the highest amount of incorporated ATRA was obtained (0.539 ± 0.005 mg) and the EE was 53.92% ± 4.98, almost the highest value obtained (Supplementary Figure S3).

Once we determined optimal ATRA loading, we modified SLN surface with PE–PEG in order to compare the drug efficiency in these different DDS. Adding 2% of PE–PEG did not significantly change SLN characteristics (Supplementary Figure S4) but slightly reduced ATRA entrapment efficiency from 54% ± 1.04 to 36% ± 4.51. Despite this reduction, total ATRA amount was high enough to study the chemotherapeutic effect.

#### 3.3.2. PEG–SLN Are More Efficient Drug Carriers than SLN without Surface Modification

**Figure 7:**
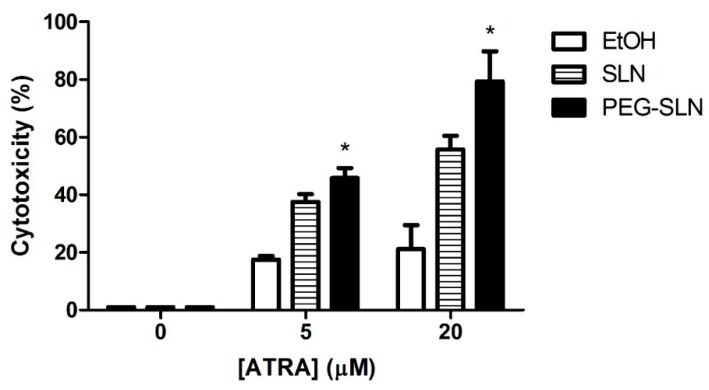
Cytotoxic effect of all-trans retinoic acid (ATRA) applied in different vehicles. SCC-25 cells were seeded (1.5 × 10^4^ cells/well) in 96-well plates. The next day, different concentrations of ATRA was added: dissolved in ethanol (empty bars), incorporated SLN (stripped bars) or incorporated PEG–SLN (black bars) and cells were incubated for 72 h. Cytotoxicity was measured using lactate dehydrogenase (LDH) method and cell viability was determined as described in Materials and Methods section. Results are expressed as the mean ± SEM of four independent experiments (* *p* < 0.05 PEG–SLN-treated values versus SLN-treated values).

We performed cell cytotoxicity experiments in order to measure the effect of ATRA dissolved in ethanol or delivered in SLN or in PEG–SLN. We observed that the cytotoxic effect of ATRA at tested concentrations (5 and 20 µM) (Figure 7) was higher when it was delivered in SLN in comparison to free ATRA. Specifically, its effect was significantly higher when ATRA was vehiculized in PEG–SLN. The most pronounced difference between cytotoxic activities of tested delivery systems was observed at 20 µM. At this concentration, free ATRA treatment showed a cytotoxicity effect of 21.16% ± 8.32 whereas SLN incorporated ATRA increased cytotoxicity to 55.74% ± 7.74 and PEG–SLN incorporated ATRA increased cytotoxicity to 79.28% ± 10.51.

## 4. Discussion

Solid lipid nanoparticles have demonstrated their ability to improve drug stability, solubility, biodistribution and efficiency. Nonetheless, although their interactions with cell membranes play a crucial role in drug delivery, remain broadly unexplored. It has been understood that internalization mechanism depends on many factors and therefore, interactions between cells and nanoparticles cannot be easily predicted. In this regard, SLN surface composition is a key factor in their cargo internalization pathway and these mechanisms should be analyzed in order to control drug delivery.

Combination of drug delivery systems with molecules to develop smart strategies to improve and drive therapeutic drugs is one of the most recurrent approaches in drug nanodelivery. In fact, this strategy is currently applied not only to nanocarriers, but also to devices and even to the therapeutic molecule itself [41,42,43]. It has been observed that selection of proper molecules of DDS improve the selected delivery of the cargo to specific organelles [44]. Therefore, smart design of the surface of nanocarriers would lead to regulation of the internalization and intracellular fate of the drug. In particular, PEG coating has been extensively applied in the development of many DDS because it improves many relevant characteristics such as stability or biocompatibility, but especially for the development of stealth DDS. Several approaches have been developed incorporating PEG to SLN in order to improve bioavailability [19,45] or to avoid immune cell recognition [46]. Unfortunately, these effects depend on different factors such as PEG size, density, conformation or anchoring molecule [22,47] and, therefore, all these parameters need to be adjusted for each particular case in order to obtain the aforementioned improvements.

In this work, we developed and characterized SLN with different amounts of PE–PEG coatings and we have observed no significant differences among their physicochemical properties. For instance, the slight reduction of ζ-potential could indicate that the surface of the SLN was only partially modified by PE–PEG as reported by other authors [48]. Nonetheless, PE–PEG presence seems to be enough to reduce SLN toxicity in a cell type dependent manner. Regarding intracellular distribution of SLN and PEG–SLN, we have not observed differences between SLN and PEG-coated SLN.

In order to analyze cell mechanisms by which SLN are incorporated we have applied most widely used pharmacological inhibitors of the main endocytosis pathways. Chlorpromazine selectively inhibits clathrin-coated pit formation [49] but it has been observed that high concentrations of this inhibitor can alter membrane characteristics [50]. Filipin inhibits caveolae-mediated endocytosis because it is a membrane disruptor that binds preferentially to cholesterol [51] and therefore it could also affect other cholesterol-dependent uptake mechanisms such as flotillin-dependent endocytosis, GTPase regulator associated with focal adhesion kinase-1 (GRAF1)-dependent endocytosis, adenosine diphosphate-ribosylation factor 6 (Arf6)-dependent endocytosis and RhoA-dependent endocytosis [52]. These endocytic pathways are relatively recently discovered mechanisms that take place in lipid rafts. Cytochalasin D inhibits actin polymerization [53], thus, inhibiting both micropinocytosis and phagocytosis. Nonetheless, it can potentially affect to other mechanisms where actin polymerization is involved, such as GRAF1-dependent endocytosis or RhoA dependent endocytosis. Thus, further experiments should be addressed in order to clearly elucidate cell internalization mechanisms and their contribution to the endocytosis of each SLN type. Interference RNA technology or combination of different pharmacological inhibitors are some of the available methods to unravel this issue [52]. The elucidation of cell internalization mechanism would clear up more details about intracellular fate of the cargo and subsequently, about the improvement of the efficiency of the delivered drug.

In our system, clathrin-mediated endocytosis was enhanced in PEG-coated SLN, suggesting that PEG-coating does not impair cell recognition. Therefore, interactions between SLN and membrane proteins are still functional, allowing active cell internalization. In our hands, PE–PEG coating does not change the interaction pattern of SLN and monocytes and cell internalization mechanisms seem to be similar for SLN and PEG–SLN. Conversely, we observed improved nanoparticle incorporation of PEG–SLN in SCC-25 cells in comparison to SLN. In this cell type, SLN were mainly incorporated by a non-active cell internalization mechanism, probably by the aforementioned unspecific “kiss-and-run” mechanism. This can be the reason why the SLN internalization is barely reduced by endocytosis inhibitors in SCC-25 cell line. Arranja and co-workers reported that PEGgylation of SLN showed altered cell internalization pattern, leading to higher therapeutic efficacy [54]. This property of PEG–SLN could be highly interesting for chemotherapeutic drugs because it is well known that cell internalization mechanism is crucial for the efficiency of the drug and for reduction of negative side effects and multidrug resistance mechanisms (i.e., P-glycoproteins).

Taking into account these promising results, more experiments should be addressed in the future to understand the reasons why this effect is so different in these cell lines. That information could shed light on the internalization mechanisms and on the influence of characteristics such as cell size and membrane topology that could be relevant factors in this context. The biased cell incorporation mechanism showed by coated or non-coated SLN led us to study the effect of surface coating on drug efficiency. We prepared ATRA loaded SLN and PEG–SLN because ATRA is a well-known chemotherapeutic agent and it has already been reported that incorporation of ATRA into SLN improves drug stability and solubility [55]. Surface modification of SLN with PE–PEG reduced slightly drug EE, an effect that was also observed by other authors [41]. Nonetheless, developed nanoparticle suspensions preset good characteristics as DDS.

Next we wanted to investigate how the PEG-coating affect the chemotherapeutic activity of ATRA. To do so cytotoxicity of ATRA-loaded PEG–SLN in SCC-25 cells was studied. Our results show that ATRA incorporated into SLN had higher cytotoxic effect than free ATRA, from 17.48% to 37.5% of cell death in the less favorable condition (5 µM of ATRA). Even more, PEG–SLN showed a striking effect on the cytotoxic effect of ATRA reaching up to 79.28% of cell death for 20 µM. This improvement allows applying lower doses of ATRA to reach equivalent antitumoral effects, alleviating side effects. In view of the changes in the cell incorporation mechanism induced by PEG surface modification, we can think that one of the reasons for this improvement on ATRA effect can rely on the different incorporation route for free or nanoparticle driven drug.

In sum, loading ATRA into SLN provides good alternatives for the use of free ATRA in chemotherapy not only because organic solvents can be avoided but also because surface modification with PE–PEG increases significantly the effect of ATRA in an oral carcinoma cell line.

## 5. Conclusions

PEG-covering did not affect physicochemical characteristics of SLN and PEG–SLN presented suitable characteristics to be applied as DDS. We have also observed that addition of PE–PEG did not impair phagocytosis of SLN by monocytic cells, but it changed interactions with cell membranes in epithelial cells, enhancing active internalization pathways. Interestingly, PEG–SLN increases all-trans retinoic acid treatment efficiency, as this formulation showed a better cytotoxicity effect than retinoic acid into SLN or in solution. In this work, we have developed an appropriate PE–PEG coating of SLN that improves the antitumoral treatment efficiency of all-trans retinoic acid. We can conclude that SLN are very versatile nanocarriers that can be tailored to achieve improvement in the efficiency of a given therapeutic treatment.

## Supplementary Materials

The following are available online at https://www.mdpi.com/2079-4991/9/3/464/s1, Figure S1: TEM micrographs of the SLN under study, Figure S2: Particle size, polydispersity index and ζ-potential values of SLN with different initial ATRA amounts, Figure S3: Determination of ATRA entrapment efficiency (EE) in different SLN suspensions and Figure S4: Particle size, polydispersity index and ζ-potential values of SLN with 1 mg of initial ATRA, with or without PE–PEG.
