# Supplementary material for: Solid Lipid Nanoparticles Surface Modification Modulates Cell Internalization and Improves Chemotoxic Treatment in an Oral Carcinoma Cell Line
Source: Nanomaterials (Basel). 2019 Mar 20;9(3):464. doi: 10.3390/nano9030464 (PMC6474192; doi:10.3390/nano9030464)
Supplement: Supplementary file 1 [file nanomaterials-09-00464-s001.pdf]

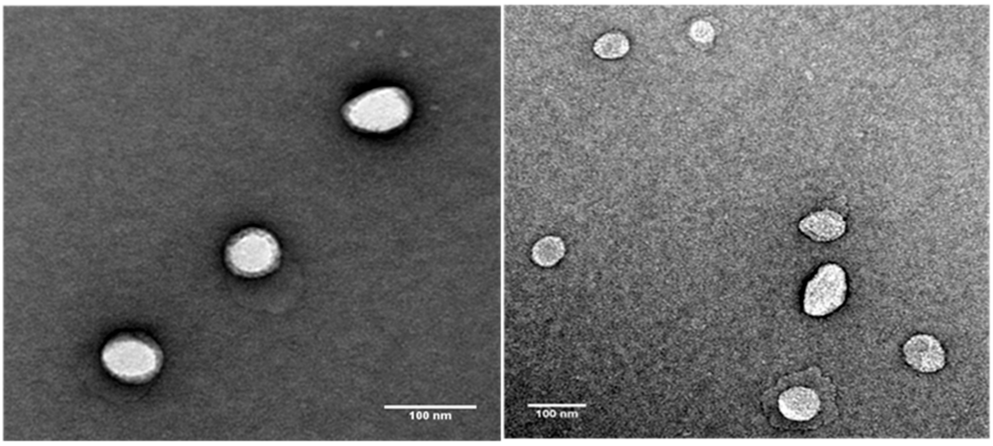


(a) (b)

**Supplementary Figure S1.** TEM micrographs of the SLN under study. A representative micrograph of non-coated SLN (**a**). A representative micrograph of 2% PEG-SLN (b). Samples were stained with uranyl acetate.

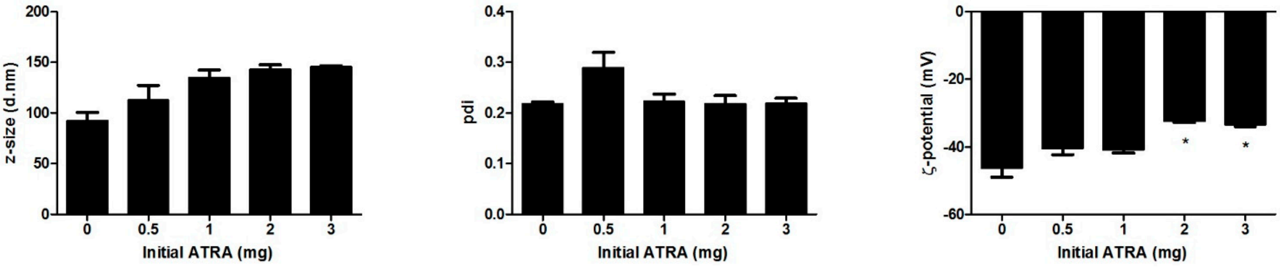


(a) (b) (c)

**Supplementary Figure S2.** Particle size, polydispersity index and ζ-potential values of SLN with different initial ATRA amounts. (a) Particle size, (b) polydispersity index and (c) and ζ–potential (C) values of different SLN were obtained by Photon Correlation Spectroscopy. Results are the mean ± SEM of four independent experiments.

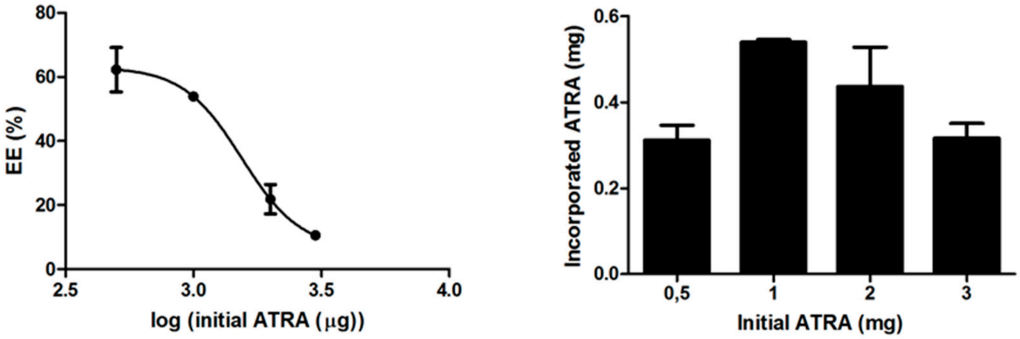


(a) (b)

**Supplementary Figure S3.** Determination of ATRA entrapment efficiency (EE) in different SLN suspensions. Indicated amount (mg) of initial ATRA was added to microemulsion mixture and SLN were obtained as described in Materials and Methods. (a) Entrapment efficiency percentage of incorporated ATRA; (b) Total amount of incorporated ATRA (mg). Results are the mean of three independent experiments.

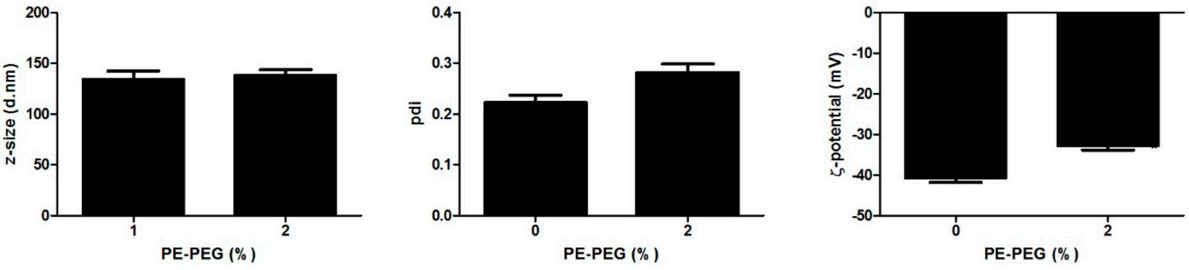


(a) (b) (c)

**Supplementary Figure S4.** Particle size, polydispersity index and ζ-potential values of SLN with 1 mg of initial ATRA, with or without PE-PEG. (a) Particle size; (b) polydispersity index and (c) ζ-potential values of different SLN were obtained by Photon Correlation Spectroscopy. Results are the mean ± SEM of three independent experiments.
